# The Anaphase-Promoting Complex or Cyclosome Supports Cell Survival in Response to Endoplasmic Reticulum Stress

**DOI:** 10.1371/journal.pone.0035520

**Published:** 2012-04-23

**Authors:** Meifan Chen, Gustavo J. Gutierrez, Ze'ev A. Ronai

**Affiliations:** Signal Transduction Program, Sanford-Burnham Medical Research Institute, La Jolla, California, United States of America; Queensland University of Technology, Australia

## Abstract

The anaphase-promoting complex or cyclosome (APC/C) is a multi-subunit ubiquitin ligase that regulates exit from mitosis and G1 phase of the cell cycle. Although the regulation and function of APC/C^Cdh1^ in the unperturbed cell cycle is well studied, little is known of its role in non-genotoxic stress responses. Here, we demonstrate the role of APC/C^Cdh1^ (APC/C activated by Cdh1 protein) in cellular protection from endoplasmic reticulum (ER) stress. Activation of APC/C^Cdh1^ under ER stress conditions is evidenced by Cdh1-dependent degradation of its substrates. Importantly, the activity of APC/C^Cdh1^ maintains the ER stress checkpoint, as depletion of Cdh1 by RNAi impairs cell cycle arrest and accelerates cell death following ER stress. Our findings identify APC/C^Cdh1^ as a regulator of cell cycle checkpoint and cell survival in response to proteotoxic insults.

## Introduction

The APC/C is a multimeric ubiquitin ligase that regulates the progression of mitosis and establishment of G1 in the cell cycle through sequential activation by the substrate-adaptors/activators Cdc20 and Cdh1 [1]. APC/C^Cdc20^ initiates anaphase and mitotic exit by targeting securin and mitotic cyclins for ubiquitination and subsequent proteasomal degradation. The switch from APC/C^Cdc20^ to APC/C^Cdh1^ in late mitosis continues the destruction of mitotic proteins including cyclin B1, Cdc20, Polo-like kinase 1 (Plk-1), and Aurora B to complete mitosis and establish G1. Sequential activation of APC/C^Cdc20^ and APC/C^Cdh1^ depends on their differential regulation by the mitotic cyclin-dependent kinases (CDKs): CDK-dependent phosphorylation of several subunits of the APC/C core promotes association with Cdc20, whereas phosphorylation of Cdh1 inhibits its binding to the APC/C core [2], [3]. This renders APC/C^Cdc20^ active in mitosis when CDK activities are high and APC/C^Cdh1^ active in telophase when CDK activities decline. Opposing the CDK-mediated inhibitory phosphorylation on Cdh1 is the phosphatase Cdc14 [3], [4]. In addition, binding of inhibitors like Emi1 (early mitotic inhibitor 1) from the G1-S transition to G2 or Rae1 (RNA export 1 homologue) in early mitosis restricts the activity of the APC/C [5], [6], [7].

The APC/C is not only a critical regulator of the cell cycle but also a key component of checkpoint signaling that can modulate cell cycle progression in response to internal and external stimuli. In an unperturbed cell cycle, APC/C^Cdc20^ is a target of the spindle assembly checkpoint (SAC), which inhibits chromosomal segregation until all sister chromatids are properly attached to the mitotic spindle [8]. Under cellular stress conditions, however, there is little evidence for checkpoint-dependent regulation of the APC/C. A few studies have reported the control of APC/C by genotoxic stress in mammalian cells. Ionizing radiation was shown to activate the APC/C to degrade cyclin D1, which triggers an immediate p53-independent G1 arrest [9]. DNA damage incurred in G2 has also been reported to activate APC/C^Cdh1^, which specifically targets Plk-1 for degradation and results in G2 arrest through the stabilization of Claspin [4], [10]. In the latter case, DNA damage-induced translocation of Cdc14B from the nucleolus to the nucleoplasm is implicated in the activation of APC/C^Cdh1^ in G2. Additionally, UV radiation triggers proteolysis of Cdh1, leading to the accumulation of cyclin B1 that promotes apoptosis [11]. While these findings from mammalian cells support the role of APC/C as an effector of checkpoint response to cellular stress, they are limited to the context of DNA damage. In contrast, APC/C^Cdh1^ in *Saccharomyces cerevisiae* is required for proper stress response to hyperosmotic shock and activation of the cell wall integrity pathway [12], prompting the question of whether cellular stresses other than DNA damage may also engage the APC/C in cell cycle checkpoint control in mammalian cells.

In this study, we explored the possible role of APC/C in regulating cell cycle response to ER stress. ER stress occurs when ER function is perturbed, which can result from physiological fluctuations in protein synthesis, pathological accumulation of misfolded proteins, or alterations in calcium levels or the redox state in the ER [13]. Three transmembrane proteins act as sensors of ER dysfunction: PERK (protein kinase RNA (PKR)-like ER kinase), IRE1 (inositol-requiring enzyme 1), and ATF6 (activating transcription factor 6). Upon detection of ER stress, the concerted action of these proteins initiate the unfolded protein response (UPR) to augment the protein folding capacity of the ER by coordinately attenuating protein synthesis through global inhibition of translation, increasing transcription of protein chaperones, and removing unfolded/misfolded proteins by transcriptional activation of regulators of ER-associated degradation (ERAD) [14], all of which serve to restore homeostasis in the ER. When this adaptive program is insufficient to restore ER function, apoptosis is often induced [15]. In addition, in mammalian cells, ER stress also activates the ER stress checkpoint to delay cell cycle progression through G1, by downregulation of cyclin D1 through PERK-mediated translational repression and proteolysis [16], [17], [18] or transcriptional induction of p21 via PERK-dependent stabilization of p53 [19]. Both the cyclin D1- and p53-dependent checkpoint responses converge at the inactivation of CDK2 by the CDK inhibitor p21 to delay G1 progression.

Intriguingly, p53-deficient HeLa cells still exhibit G1 arrest in response to ER stress that cannot be overcome by the overexpression of cyclin D1 [20], indicating the presence of p53- and cyclin D1-independent pathways in the control of the ER stress checkpoint. We recently identified one such pathway involving the accumulation of p27, also an inhibitor of CDK2, as a result of reduced deubiquitination and transcriptional downregulation of Skp2 [20]. Our findings also suggested the existence of additional cell cycle components that contribute to the ER stress checkpoint.

Here, we identify APC/C^Cdh1^ activity as a key component of the p53- and cyclin D1-independent ER stress checkpoint pathway that delays cell cycle progression through G1. ER stress-dependent activation of APC/C^Cdh1^ is required to coordinate cell cycle delay, and depletion of Cdh1 sensitizes cells to apoptosis under ER stress conditions. Collectively, our findings not only indicate that APC/C^Cdh1^ is a regulator of checkpoint signaling and cell viability in response to proteotoxicity, while unveiling the potential of the APC/C to mediate a range of stress-induced checkpoint responses wider than previously anticipated.

## Results

### APC/C^Cdh1^ is activated under ER stress conditions

**Figure 1:**
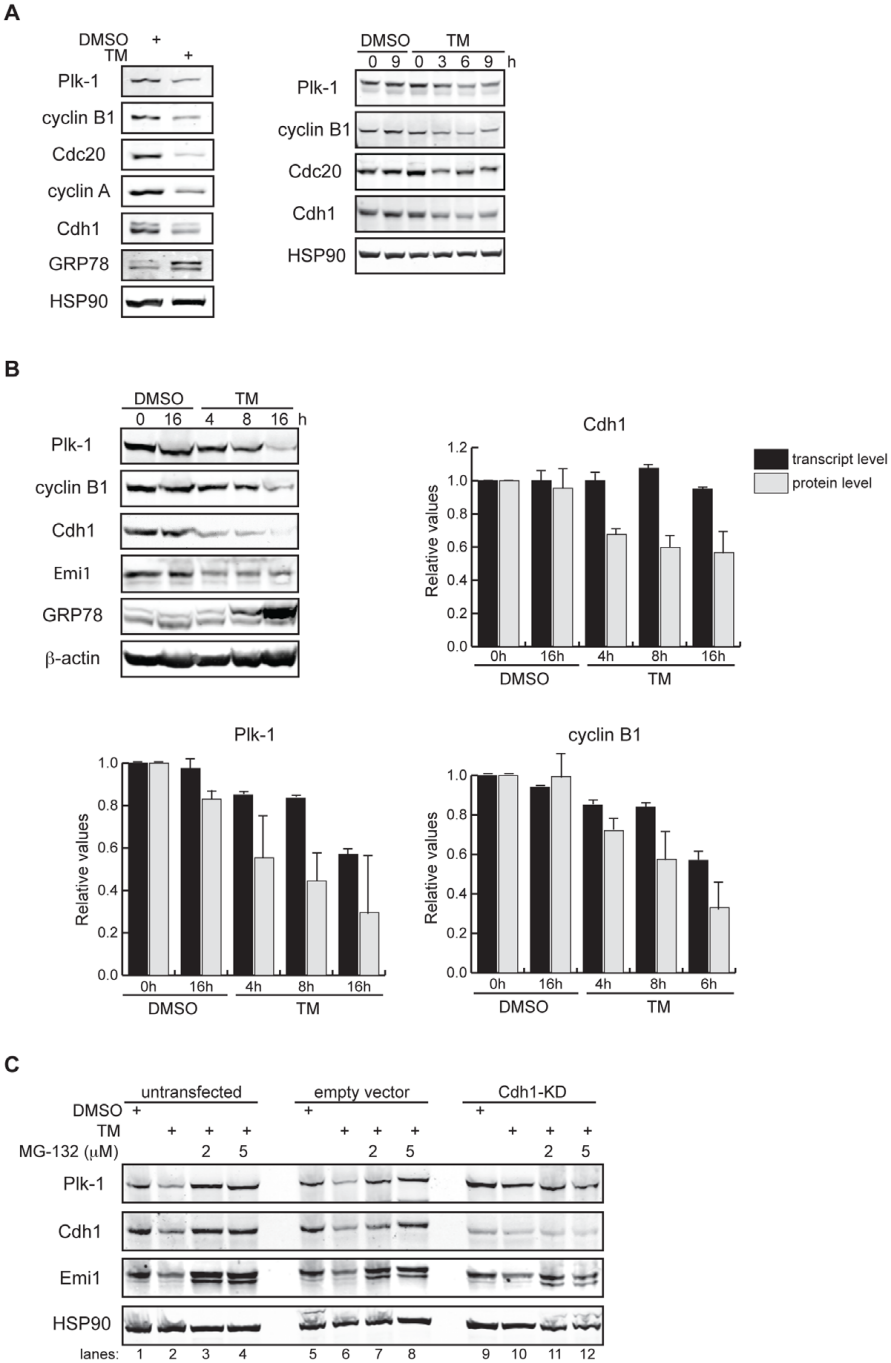
APC/C^Cdh1^ is activated under ER stress conditions. (A) *Left*, HeLa cells were treated with DMSO or 2.5 µg/ml of tunicamycin (TM) for 8 h. Total cell lysates were immunoblotted for the indicated endogenous proteins. GRP78 served as a marker of ER stress. *Right*, HeLa cells were harvested at the indicated times after addition of DMSO or 2.5 µg/ml of TM. Total cell lysates were immunoblotted for the indicated endogenous proteins. HSP90 was used as a loading control. (B) HeLa cells were treated with DMSO or 1 µg/ml of tunicamycin for the indicated times. *Immunoblots*, total cell lysates were immunoblotted for the indicated endogenous proteins. *Graphs*, transcript levels as measured by SYBR-green qRT-PCR and protein levels as quantified by LiCOR-Odyssey software on immunoblots are compared for the indicated proteins. All measurements were normalized to the DMSO-0 h sample with relative value of 1. (C) HeLa cells were transfected with pSUPER (empty vector) or pSUPER-Cdh1-shRNA (Cdh1-KD). 24 h after transfection, cells were treated with DMSO, 1 µg/ml of TM alone, or 1 µg/ml of TM plus MG-132 (2 or 5 µM) for 16 h. Total cell extracts were immunoblotted for the indicated endogenous proteins.

When we analyzed the cell cycle response to ER stress in HeLa cells by treatment with tunicamycin (TM), a pharmacological ER stress inducer that inhibits glycosylation of newly synthesized glycoproteins, we observed a marked reduction in the protein levels of endogenous cyclins A and B1, Cdc20, and Plk-1 –all of which are known substrates of the APC/C^Cdh1^ [1] (Figure 1A). To determine whether such changes in expression occur transcriptionally or post-translationally, we compared the kinetics of downregulation at the mRNA and protein levels. We found that while transcriptional repression likely participated in the reduction of APC/C^Cdh1^ substrates under prolonged TM treatment (Figures 1B-bottom graphs and S1), protein downregulation occurred at a faster rate than transcriptional repression of these substrates, as observed at the early time points following TM treatment (Figures 1A-right panel and 1B). These results suggest that ER stress downregulates APC/C^Cdh1^ substrates both by protein degradation and transcriptional repression. The ability of the proteasome inhibitor, MG-132, to block TM-dependent downregulation of Plk-1 further supports accelerated protein degradation of APC/C^Cdh1^ substrates following ER stress (Figure 1C, lanes 1–4). The TM-dependent decrease of the cell cycle proteins examined here was selective and not due to a general increase in proteasomal activity, as the protein stability of the cell cycle regulator p27 is prolonged by TM treatment [20]. Taken together, TM-induced protein destabilization of APC/C^Cdh1^ substrates suggests activation of APC/C^Cdh1^ under ER stress conditions.

To determine whether APC/C^Cdh1^ is responsible for the degradation of its substrates under ER stress conditions, we next tested if depletion of Cdh1 could overcome TM-dependent downregulation of its substrates. Cdh1 knockdown by RNAi (Cdh1-KD) alone attenuated TM-induced reduction of Plk-1 (Figure 1C, lanes 9–12; & data below) and cyclin A (see data below), supporting the hypothesis that APC/C^Cdh1^ is activated following ER stress induction. Depletion of Cdh1, however, did not fully reverse downregulation of its substrates probably due to TM-dependent transcriptional repression.

Curiously, TM treatment also reduced Cdh1 expression (Figures 1A and 1B) solely by protein degradation, as no change in the transcription of Cdh1 (Figures 1B and S1) but rather a steady decrease in both the endogenous and exogenous Cdh1 protein levels was observed (Figures 1B and S2A, respectively). In addition, MG-132 restored Cdh1 protein levels upon TM treatment (Figure 1C, lanes 1–4). To test whether ER stress triggers auto-ubiquitination of Cdh1 by APC/C^Cdh1^ [21], we depleted the APC/C core subunit —Cdc27/APC3— by RNAi and examined its effect on TM-induced downregulation of Cdh1. To our surprise, knockdown of Cdc27 did not restore the levels of Cdh1 following TM treatment (Figure S2B), suggesting that while TM treatment enhances APC/C^Cdh1^ activity, it also induces degradation of Cdh1 by an ubiquitin ligase other than the APC/C.

### Depletion of Cdh1 overcomes ER stress-induced G1 delay

**Figure 2:**
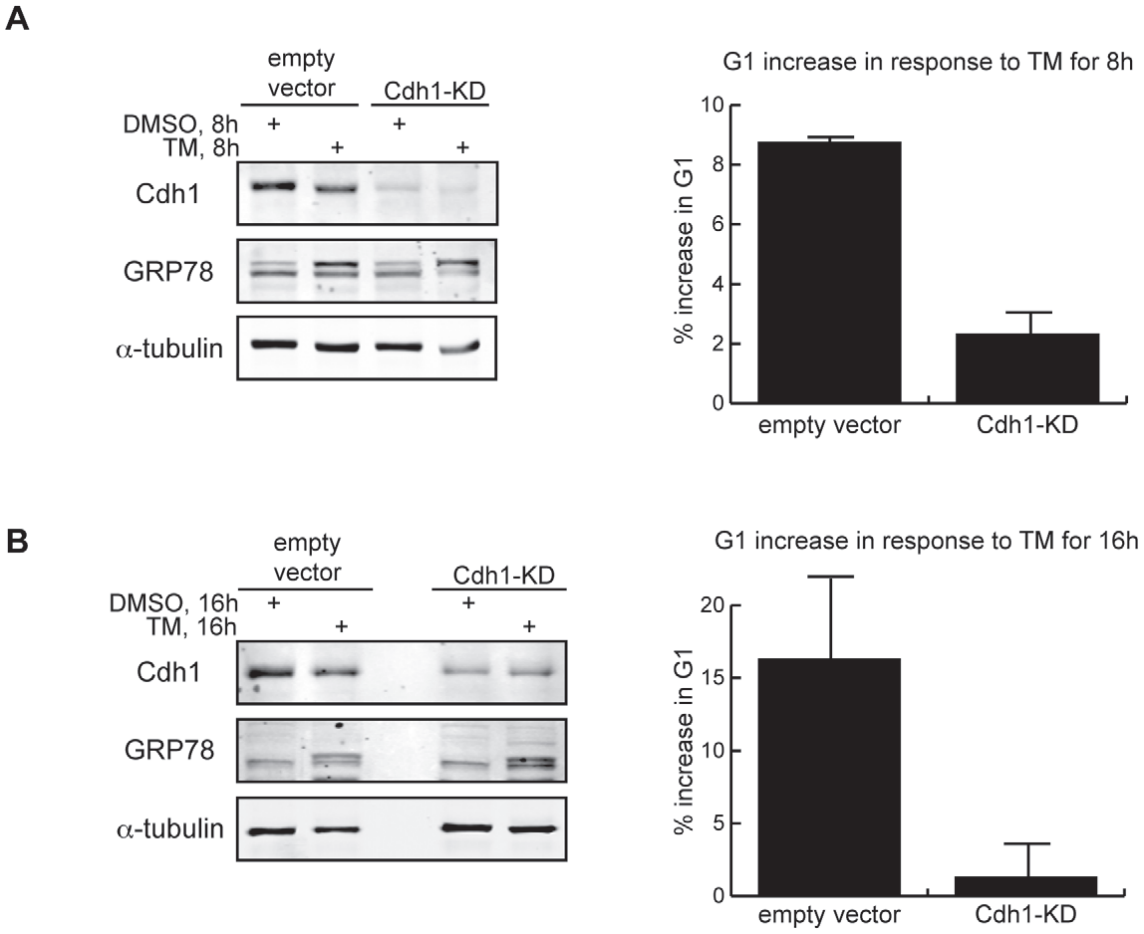
Depletion of Cdh1 overcomes ER stress-induced G1 delay. (A) Empty vector-transfected and Cdh1-KD HeLa cells were treated with DMSO or 2.5 µg/ml of TM for 8 h. *Left*, knockdown efficiency was verified by immunoblotting of endogenous Cdh1. *Right*, cell cycle distribution of the same cells was analyzed by FACS. Graph quantifies the increase in percentage of cells in G1 after treatment with 2.5 µg/ml of TM for 8 h, normalized to the percentage in DMSO-treated samples. (B) Empty vector-transfected and Cdh1-KD cells were then treated with DMSO or 1 µg/ml of TM for 16 h. *Left*, knockdown efficiency was verified by immunoblotting of endogenous Cdh1. *Right*, graph quantifies the increase in percentage of cells in G1 after treatment with 1 µg/ml of TM for 16 h, normalized to the percentage in DMSO-treated samples.

Having shown that ER stress activates APC/C^Cdh1^, we next sought to determine whether APC/C^Cdh1^ activity is required for the cell cycle response to ER stress, in particular, the G1 ER stress checkpoint [17], [20]. We treated asynchronous control-transfected or Cdh1-KD cells with TM for 8 h or 16 h and analyzed cell cycle distribution by fluorescence activated cell sorting (FACS). ER stress delayed progression through G1 in a time-dependent manner (Figures 2A-graph, 2B-graph, S3A, and S3B) as previously shown [17], [20]. Depletion of Cdh1 largely overcame this effect, reducing the TM-induced G1 increase from 8 and 15% at 8 h and 16 h respectively in control cells to 1 and 3% in the Cdh1-KD cells (Figures 2A, 2B, and S3A and S3B). These findings support the requirement for APC/C^Cdh1^ activity in the establishment and/or maintenance of ER stress-dependent G1 delay.

### Mechanisms supporting APC/C^Cdh1^ activity under ER stress

**Figure 3:**
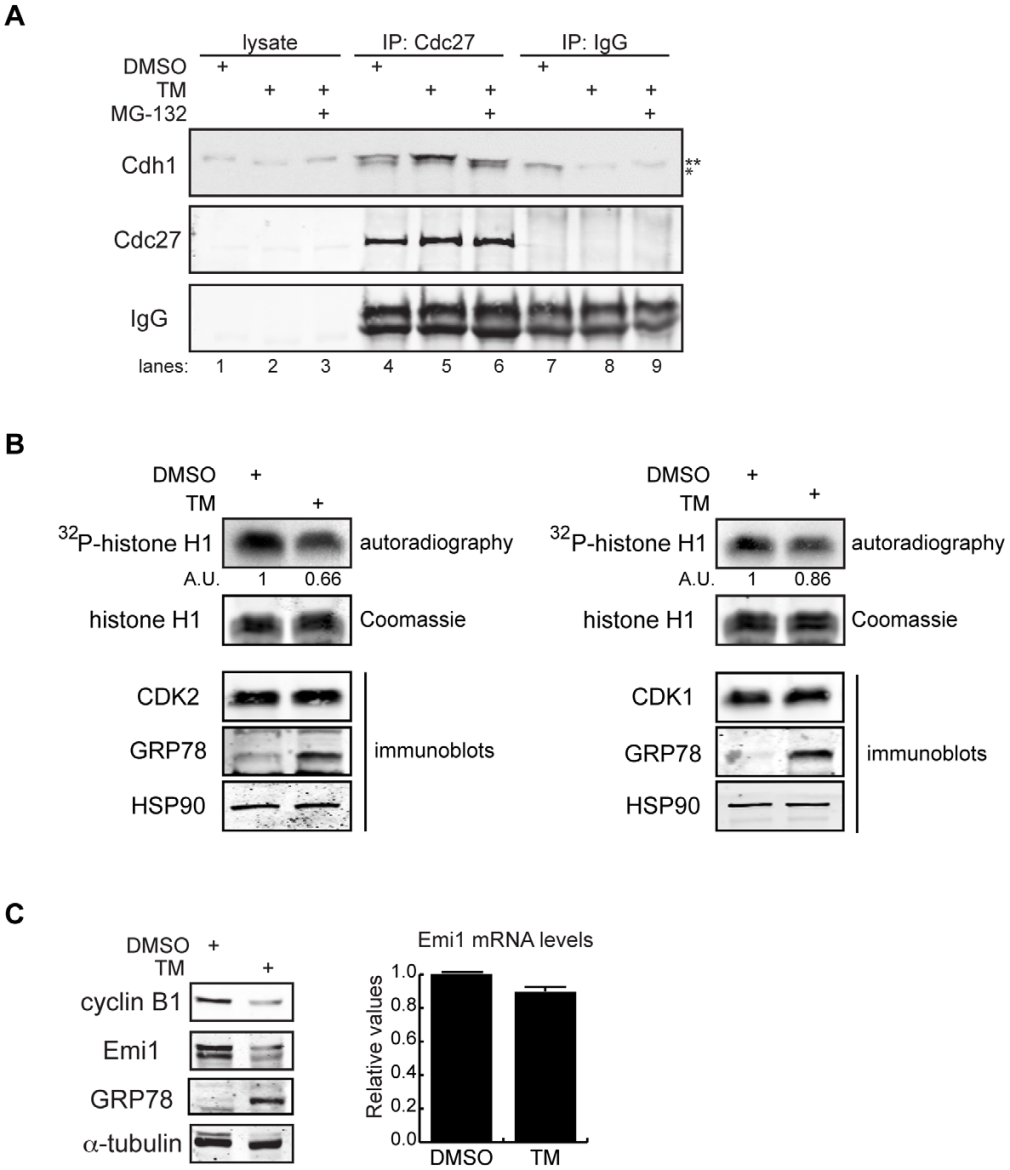
Mechanisms supporting APC/C^Cdh1^ activity under ER stress conditions. (A) HeLa cells were treated with DMSO, 0.5 µg/ml of TM alone, or 0.5 µg/ml of TM plus 5 µM of MG-132 for 16 h. Immunoprecipitates of endogenous Cdc27 were immunoblotted for endogenous Cdh1. Immunoprecipitation using IgG served as negative control. ** indicates Cdh1-specific top band; *indicates non-specific bottom band. (B) HeLa cells were treated with DMSO or 1 µg/ml of TM for 16 h. CDK2 or CDK1 antibodies were used to immunoprecipitate endogenous CDK2 or CDK1 complexes, respectively. Immunoprecipitates were then used in *in vitro* kinase assays using histone H1 as substrate. *Left*, autoradiography of ^32^P-histone H1 phosphorylated by CDK2 complexes. Coomassie stains input of histone H1 in the reactions. Intensity of the autoradioactive bands were quantified by Image J, normalized to histone H1 input, and presented as arbitrary units (A.U.). Immunoblot shows comparable levels of endogenous CDK2 and the indicated proteins before and after TM treatment. *Right*, autoradiography of ^32^P-histone H1 phosphorylated by CDK1 complexes, analyzed as indicated for CDK2. (C) *Left*, total cell lysates from HeLa cells treated with DMSO or 1 µg/ml of TM for 16 h were immunoblotted for the indicated endogenous proteins. *Right*, quantification of endogenous Emi1 mRNA levels in HeLa cells treated with DMSO or 1 µg/ml of TM for 16 h, measured by SYBR-green qRT-PCR.

To explore possible mechanisms that contribute to APC/C^Cdh1^ activation following TM treatment, we examined several parameters. First, because complex formation between the APC/C core and Cdh1 is a prerequisite for APC/C^Cdh1^ activation, we tested whether ER stress promotes APC/C^Cdh1^ assembly. Surprisingly, although TM reduced Cdh1 protein levels (Figure 1A), it enhanced the association between endogenous Cdh1 and the APC/C core subunit Cdc27/APC3 (Figure 3A, compare lanes 4 and 5) concomitant with induction of G1 arrest (Figure S4A). Addition of MG-132 reversed the effect of TM on APC/C^Cdh1^ assembly (Figure 3A, compare lanes 4–6), despite the fact that MG-132 treatment stabilized the Cdh1 protein (Figures 1C and 3A). The inhibitory effect of MG-132 on complex formation of APC/C^Cdh1^ likely results from its ability to arrest the cell cycle in G2/M (Figures S4B and S4C), in which high mitotic Cdk activity blocks Cdh1 binding to the APC/C core [3], [22], [23]. This observation reveals that the Cdh1 protein level does not always positively correlate with the abundance of active APC/C^Cdh1^ complexes.

Since CDK-dependent phosphorylation of Cdh1 inhibits the assembly of APC/C^Cdh1^, in part by localizing Cdh1 to the cytoplasm, we next examined CDK1 and CDK2 activities in TM-treated HeLa cells. Using *in vitro* kinase assays, we found both activities to be reduced by TM treatment (Figure 3B), suggesting that reduced inhibitory phosphorylation of Cdh1 may also contribute to promote APC/C^Cdh1^ complex formation following induction of ER stress.

Furthermore, APC/C is also controlled by association with proteins that inhibit its activation and/or assembly. Emi1 is an APC/C inhibitor that acts by competitive binding to APC/C^Cdh1^ as a pseudosubstrate [5], [6]. The transcription factor E2F induces Emi1 transcription at the G1-S transition, which results in inhibition of APC/C^Cdh1^ that promotes G1 exit [24]. Upon examining the expression of Emi1, we found that TM treatment drastically decreased the protein levels of Emi1 (Figures 1C and 3C, *Left*). This was not a result of transcriptional repression (Figure 3C, *Right*), but rather a result of proteasomal degradation that could be rescued by MG-132 (Figure 1C, lanes 1–4). To test the possibility that ER stress conditions may convert Emi1 from a pseudosubstrate into a *bona fide* substrate of APC/C^Cdh1^, we examined the effect of TM on Emi1 protein expression in Cdh1-depleted cells. We observed no difference in the degree of ER stress-dependent Emi1 downregulation between control and Cdh1-KD cells (Figures 1C, lanes 9–10 and S5), arguing against a role for the APC/C^Cdh1^ in Emi1 degradation under ER stress conditions. Finally, overexpression of Emi1 partially blocked the TM-induced downregulation of APC/C^Cdh1^ substrates (Figure S6), suggesting that ER stress-dependent Emi1 downregulation promotes APC/C^Cdh1^ activity. Taken together, cellular ER stress ensures APC/C^Cdh1^ activation through multiple mechanisms including enhanced affinity between Cdh1 and subunits of the APC/C core, reduced activity of CDKs, and Emi1 downregulation. Although the mechanisms examined above support APC/C^Cdh1^ activity, it is unclear whether they are the initiating events that activate APC/C^Cdh1^ in response to ER stress or the consequence of ER stress-induced G1 phase delay.

### Cdh1 depletion sensitizes cells to ER stress-induced cell death

**Figure 4:**
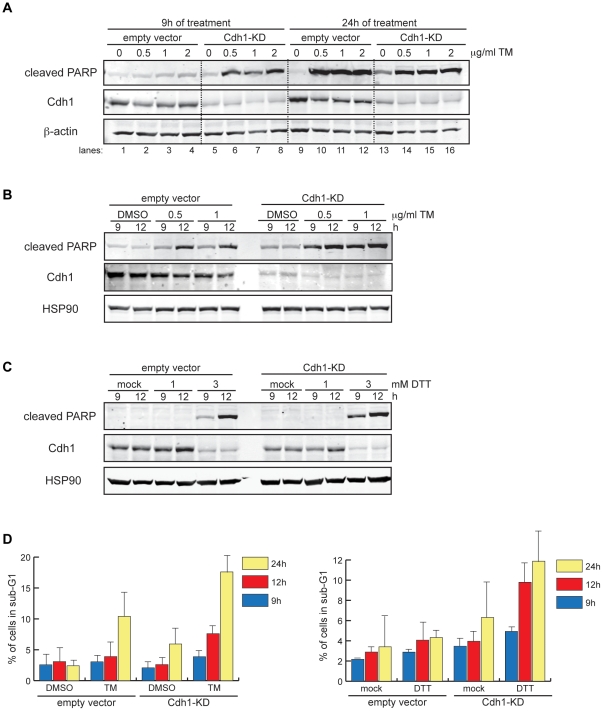
Cdh1 depletion sensitizes cells to ER stress-induced cell death. (A) Empty vector-transfected or Cdh1-KD cells were treated with DMSO or 0.5, 1, or 2 µg/ml of TM for 9 h or 24 h. Total cell lysates were immunoblotted for cleaved PARP and indicated endogenous proteins. (B) Empty vector-transfected or Cdh1-KD cells were treated with DMSO, 0.5 µg/ml, or 1 µg/ml of TM for 9 h or 12 h. Total cell lysates were analyzed as in (A). (C) Empty vector-transfected or Cdh1-KD cells were treated with solvent (mock) or 1–3 mM DTT for 9 h or 12 h. Total cell lysates were immunoblotted as in (A). (D) *Left*, empty vector-transfected or Cdh1-KD cells were collected at 9, 12, and 24 h after treatment with DMSO or 0.5 µg/ml of TM for DNA content analysis by flow cytometry. Graph shows percentage of sub-G1 population at each time point. *Right*, empty vector-transfected or Cdh1-KD cells were collected at 9, 12, and 24 h after treatment with solvent (mock) or 1 mM DTT for DNA content analysis by flow cytometry. Graph shows percentage of sub-G1 population at each time point.

APC/C^Cdh1^ has been reported to target the ER stress-induced pro-apoptotic proteins c-Jun N-terminal kinase (JNK) and tribbles-related protein 3 (TRB3) for ubiquitin-proteasome degradation [25], [26], [27], [28]. Our finding that APC/C^Cdh1^ becomes active following ER stress induction raises the possibility that APC/C^Cdh1^ may exert a pro-survival effect under such conditions. To test this, we compared the susceptibility of control and Cdh1-KD cells to TM-induced cell death upon TM treatment by immunoblotting for cleaved PARP, a marker of apoptosis resulting from cleavage by the pro-apoptotic protease caspase 3 [29], [30]. Cleaved PARP was strongly induced by 24 h after TM treatment in both control-transfected and Cdh1-KD cells, but was detectable as early as 9 h after treatment only in Cdh1-KD cells (Figure 4A). Because prolonged Cdh1-KD adversely affected basal cell viability (Figure 4A, compare lanes 9 and 13), we compared cell survival of TM-treated control and Cdh1-KD cells at the shorter time-point of 12 h and again found increased sensitivity of Cdh1-KD cells to apoptosis at 9 h and 12 h of treatment (Figure 4B). Enhanced susceptibility to cell death was also seen in Cdh1-KD cells after treatment with another ER stress inducer, the disulfide bond-reducing agent dithiothreitol (DTT) (Figure 4C).

Additionally, we measured the DNA content by flow cytometry in order to quantify apoptotic cells, which have diminished DNA content and appear as a sub-G1 population. Quantification of the sub-G1 population showed a more than two-fold increase in cell death in Cdh1-KD compared with the control cells at 12 h of TM treatment (Figure 4D, representative FACS histograms in Figure S7). Taken together, these data show that Cdh1-KD accelerates ER stress-induced cell death between 9 h and 24 h after TM treatment; however, extended depletion of Cdh1 alone compromises overall cell survival. A similar pattern of cell death was found after DTT treatment (Figure 4D).

**Figure 5:**
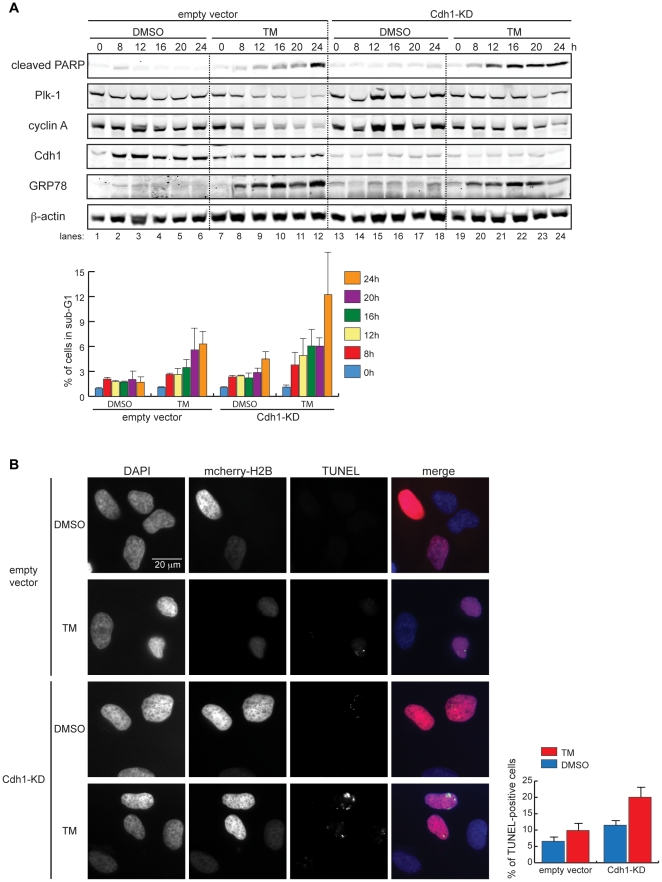
Cdh1 depletion sensitizes cells to ER stress-induced cell death. (A) *Top*, empty vector-transfected or Cdh1-KD cells were collected at 0, 8, 12, 16, 20, and 24 h after treatment with DMSO or 1 µg/ml of TM. Total cell lysates were immunoblotted for the indicated proteins. DNA content of these cells was analyzed by flow cytometry. *Bottom*, graph shows percentage of sub-G1 population at each time point. (B) HeLa cells were co-transfected with empty vector plus mcherry-H2B, or Cdh1-shRNA plus mcherry-H2B. 24 h after transfection, DMSO or 1 µg TM was added to cells for 16 h. Fluorescence microscopy was used to visualize the nuclei (by DAPI staining), transfected cells (by mcherry-H2B), and apoptosis-induced DNA strands breaks (by TUNEL staining). *Bottom* graph quantifies TUNEL-positive cells.

In a more detailed comparison of the kinetics of TM-induced cell death in control and Cdh1-KD cells, we again observed enhanced apoptosis in Cdh1-KD cells between 12 h and 24 h of treatment, with evident PARP cleavage in Cdh1-KD cells but not in the control (Figure 5A, *Top* immunoblot panels), and increased sub-G1 population in Cdh1-KD cells (Figure 5A, *Bottom* graph). Notably, the protein expression of APC/C^Cdh1^ substrates such as Plk-1 and cyclin A was prolonged in TM-treated Cdh1-KD cells compared to the TM-treated control (Figure 5A, *Top* panels).

Finally, examination of DNA strand breaks by TdT-mediated dUTP nick end labeling (TUNEL) revealed that twice as many TM-treated Cdh1-KD cells were TUNEL-positive as TM-treated control cells (Figure 5B), consistent with our findings from detection of apoptosis based on PARP cleavage and sub-G1 quantification. Taken together, the concurrent deregulation of cell cycle response and sensitization to cell death in Cdh1-KD cells under ER stress conditions suggest a pro-survival role for the APC/C^Cdh1^-dependent ER stress checkpoint.

We next sought to identify downstream effector(s) of ER stress-induced apoptosis in the absence of Cdh1. JNK and the CDK1-activating subunit cyclin B1 are two appealing candidates because both are substrates of the APC/C^Cdh1^ [28], [31] with pro-apoptotic functions [32], [33]. To examine this, control and Cdh1-KD cells were monitored for cell death by PARP cleavage and DNA content after co-treatment with TM and either the JNK inhibitor SP600125 [34] or the pan-CDK inhibitor roscovitine [35] for 8, 12, and 24 h. We found increased cell death in both cell types at all time points after co-treatment of TM and SP600125, compared with TM treatment alone (Figure S8, *Left* graph). In contrast, co-treatment with roscovitine reduced cell death of both control and Cdh1-KD cells by almost 50% at 24 h, but not at earlier time-points (Figure S8, *Right* graph). These results point to the involvement of CDKs in mediating cell death under prolonged ER stress conditions (24 h in TM). However, the inability of roscovitine to rescue cell death at earlier time points, when Cdh1-KD specifically enhances TM-dependent apoptosis, suggests that the activity from CDKs is not responsible for the accelerated ER stress-induced cell death in the absence of Cdh1 (Figure S8). Altogether, these data suggest that neither JNK nor CDKs mediate the death of Cdh1-KD cells under ER stress conditions.

## Discussion

Our study provides evidence for activation of APC/C^Cdh1^ under ER stress conditions with concomitant downregulation of the APC/C inhibitor Emi1 and enhanced interaction between Cdh1 and the APC/C core subunits. At present, it is not clear whether these events activate APC/C^Cdh1^ in response to ER stress to initiate cell cycle arrest or are a consequence of ER stress-induced cell cycle delay. In either case, the activity of APC/C^Cdh1^ was found to positively regulate the ER stress checkpoint: depletion of Cdh1 by RNAi partially rescued TM-induced degradation of APC/C^Cdh1^ substrates as well as G1 delay. It remains to be determined whether the activity of APC/C^Cdh1^ is involved in the establishment or maintenance of such cell cycle response.

Our observations that TM triggers protein destabilization of Cdh1 and Emi1 raise the question of what ubiquitin ligases target them for degradation under such conditions. It is also formally possible that ER stress translationally represses Cdh1 and Emi1.

The requirement for APC/C^Cdh1^ in the engagement of the ER stress checkpoint is independent of that for p53 and cyclin D1, but reinforces the previously identified Ufd1-USP13-Skp2-p27 axis [20]: as the decline in Ufd1 reduces the deubiquitination of Skp2, APC/C^Cdh1^ is concurrently activated to ubiquitinate Skp2 and its other substrates. However, the cyclin D-, p53-, and APC/C^Cdh1^-dependent pathways may not be mutually exclusive in establishing and/or maintaining the ER stress checkpoint, as the downregulation of APC/C^Cdh1^ substrates at the protein level was also detected in normal human foreskin fibroblasts with intact p53 (Figure S9). This suggests establishment of the ER stress checkpoint through the coordinated regulation of multiple cell cycle pathways.

In addition to bypassing the checkpoint response, Cdh1-depleted cells were more sensitive to ER stress-induced apoptosis, underscoring the pro-survival function of APC/C^Cdh1^ under such conditions and supporting the G1 ER stress checkpoint as a pro-survival mechanism to cope with proteotoxic conditions. The latter implication is consistent with our previous finding that G1 phase of the cell cycle promotes degradation of ERAD substrates [20]. If it is halting cell cycle progression that is critical for cell survival under ER stress, then it is likely that induction of the entire degradation program of APC/C, rather than degradation of any single substrate, is required for viability in response to ER stress.

Prolonged ER stress induces apoptosis through IRE1 and CHOP (C/EBP homologous protein) by mechanisms that include IRE1-dependent recruitment of TRAF2 (tumor necrosis factor receptor-associated factor 2) leading to activation of JNK [36] and CHOP-dependent suppression of the pro-survival protein Bcl-2 [37]. Here, we describe previously unrecognized roles for cell cycle proteins in the regulation of ER stress-induced cell death: the APC/C^Cdh1^ that provides initial protection against apoptosis and the cyclin-CDK activities that contribute to cell death under prolonged ER stress conditions.

There are several implications for the identification of the APC/C^Cdh1^ as a key regulator of checkpoint response and cell survival under ER stress. First, the repertoire of stress conditions that engage the APC/C^Cdh1^ in coordinating the cell cycle response is no longer limited to genotoxic stress in mammalian cells. Indeed, based on our findings, any condition that disrupts ER homeostasis may regulate APC/C^Cdh1^, including alterations in metabolic conditions, redox states, and/or intracellular calcium concentrations [38]. Second, the pro-survival role of APC/C^Cdh1^ under ER stress conditions first identified here suggests that APC/C^Cdh1^ may act as a switch that determines cell fate in response to ER stress. Finally, given the widely recognized link between chronic ER stress-induced apoptosis and human diseases such as neurodegeneration and diabetes [39], our current findings suggest that cell cycle proteins may be effectors in the pathogenesis of these diseases and possible therapeutic targets for diseases characterized or accompanied with ER stress.

## Materials and Methods

### Cell culture and transfection

HeLa [human cervical adenocarcinoma epithelial] cells [27] were grown in DMEM with 10% bovine serum at 37°C with 5% CO2. Human foreskin fibroblasts (HFF-1) were grown in DMEM with 10% fetal bovine serum. All cell lines were obtained from the American Type Culture Collection (ATCC) and tested free of mycoplasma during the complete experimental phase of the project. For transient transfection of plasmids in HeLa cells, 80%-confluent cells grown in 10 cm dishes were transfected with 5 µg of each of the indicated plasmids, using Lipofectamine 2000 (Invitrogen) following manufacturer's protocols.

### Expression plasmids

pSUPER-empty vector and pSUPER-Cdh1-shRNA were generous gifts from Dr. Reuven Agami (The Netherlands Cancer Institute, Amsterdam, Netherlands). Both pCS2+-myc empty vector and pCS2+-myc-Emi1 were generous gifts from Dr. Peter K. Jackson (Genentech, San Francisco, USA). pCMV-myc-Cdh1 was used to overexpress Cdh1 in HeLa cells [28].

### shRNA target sequence

pSUPER-Cdh1-shRNA: TGAGAAGTCTCCCAGTCAG – previously published [40].

### Antibodies

Commercially available antibodies used in this study were: Cdh1 (DCS-266– Abcam at 1∶1000 dilution), cyclin B1 (GNS1– Santa Cruz at 1∶1000), cyclin A (H-3– Santa Cruz at 1∶1000), GRP78 (N-20– Santa Cruz at 1∶1000), c-myc (9E10– Santa Cruz at 1∶2000), Plk-1 (F-8– Santa Cruz at 1∶1000), Cdc20 (ab26483– Abcam at 1∶500), CDK1 (SC-54– Santa Cruz at 1∶1000), CDK2 (SC-163– Santa Cruz at 1∶1000), cleaved PARP (9541– Cell Signaling Technology at 1∶1000), Emi1 (37-6600– Invitrogen at 1∶500), Cdc27 (AF3.1– Santa Cruz at 1∶1000), HSP90 (F-8– Santa Cruz at 1∶1000), α-tubulin (TU-02– Santa Cruz at 1∶2000).

### Immunoblotting and immunoprecipitation

For western blots of total cell lysates, cells were detached from plates with 0.05% trypsin-EDTA, washed with cold PBS, resuspended in IP buffer (50 mM Tris-HCl [pH = 7.6], 150 mM NaCl, 0.5% NP-40, 5 mM EDTA, 5 mM EGTA, 20 mM NaF, 100 µM sodium-orthovanadate, 2 mM β-glycerophosphate, 1 mM DTT, 1 mM PMSF, 4 mg/ml aprotinin, 100 µM leupeptin, and 2 mg/ml pepstatin A), and lysed on ice for 20 min. Lysates were then centrifuged for 20 min (13200 rpm at 4°C), supernatants were saved, and protein concentration was performed using Coomassie Plus Protein Assay Reagent (Thermo Scientific) following manufacturer's recommendations.

For *in vivo* co-immunoprecipitation, cells were washed with cold PBS and lysed with IP buffer. Target protein was immunoprecipitated from total cell lysates containing 2 mg of protein with 1 µg of target-specific antibody overnight at 4°C in a rotating wheel, followed by incubation with 30 µl of 50% slurry of protein G-agarose for 2 h at 4°C with rotation. Immunoprecipitates were washed 4 times with IP buffer, resuspended in loading buffer and analyzed by SDS-PAGE and western blotting.

### In vitro kinase assay

Cells were lysed with modified IP buffer (0.1% NP-40, 25 mM Tris-HCl [pH = 7.6], 50 mM NaCl, 5 mM EGTA, 60 mM β-glycerophosphate, 20 mM NaF, 100 µm sodium-orthovanadate, 1 mM DTT, 1 mM PMSF, 100 µM leupeptin, and 4 mg/ml aprotinin). Endogenous CDK1 and CDK2 were immunoprecipitated from approximately 400 µg of total cell lysate, using 0.5 µg of CDK2- or CDK1-specific antibody. Immunoprecipitates were washed 3 times with histone H1 buffer (15 mM MgCl2, 20 mM EGTA, 80 mM β-glycerophosphate pH 7.4, 1 mM DTT, 1 mM PMSF, 4 mg/ml aprotinin, and 100 µM leupeptin). Kinase reactions were performed by incubating pellets with 35 µl of histone H1 buffer supplemented with 0.1 µg histone H1, 0.2 µl ^32^P-γATP and 50 µM unlabeled ATP. Reactions were incubated for 30 min at 30°C in shaking at 800 rpm and then stopped by addition of SDS-PAGE sample buffer and heat-denaturation. Samples were analyzed by SDS-PAGE. Gels were Coomassie-stained, dried, and exposed to film.

### DNA content analysis by fluorescence-activated cell sorting (FACS)

Cells were washed with cold PBS, fixed with 70% ethanol in PBS for 1 h at −20°C, and washed again with cold PBS. Cells were then incubated in PBS containing 50 µg/ml propidium iodide (PI) and 100 µg/ml RNase A for 1 h at room temperature. Analysis of DNA content was performed on FACSCanto, using 488 nm excitation. FlowJo and ModFit LT software were used to analyze cell cycle profile.

For detection of apoptotic cells with reduced DNA content by FACS, cells were fixed in ethanol as described above, then subjected to mild extraction of low molecular weight DNA by incubating with 0.2 M phosphate citrate buffer [pH7.8] for 30 min at 37°C on a shaker. Cells were then centrifuged and stained with PI as described above.

### TdT-mediated dUTP nick end labeling (TUNEL)

HeLa cells were seeded on coverslips and co-transfected with 6 µg of pSUPER empty vector plus 1 µg of mcherry-H2B, or 6 µg of pSUPER-Cdh1-shRNA plus 1 µg of mcherry-H2B for 24 h. Co-transfection with mcherry-H2B enables monitoring of transfection efficiency by fluorescence microscopy. Cells were then treated with DMSO or 1 µg/ml TM for 16 h, washed with PBS twice, fixed in 4% paraformaldehyde with 2% sucrose in PBS for 20 min at room temperature. Detection of apoptosis-induced DNA strand breaks was performed according to the manufacturer's protocol using the *In Situ* Cell Death Detection Kit, AP (Roche Applied Sciences), followed by 4′,6-diamidino-2-phenylindole (DAPI) staining of the nuclei. Fluorescein-labeled DNA strand breaks were examined under a fluorescence microscope with an excitation wavelength of 450–500 nm (green). 300 mcherry-H2B-positive cells were counted for quantification of TUNEL-positive cells under each condition.

### SYBR-green qRT-PCR

Total RNA was extracted from cells grown in 10 cm plates (Sigma-Aldrich, RTN70-1KT). Up to 2 µg of total RNA was used per 20 µl cDNA reverse transcription reaction (Applied Biosystems, High-Capacity cDNA Reverse Transcription Kit). A 5 µl aliquot of 20% (vol/vol) diluted cDNA was used per 25 µl of qRT-PCR with SYBR GreenER qPCR SuperMix Universal (Invitrogen) and gene-specific primers. Forty cycles of qPCR and analysis were performed using Stratagene Mx3000p. Human cyclophilin was used as a control to normalize transcript levels of experimental samples. The specificity of all primer sets was validated by dissociation curves. qPCR primer sequences were as follows:

**Cdh1.**

Forward - GCAACAAGAGCCAGAAGCTGC

Reverse - CCAGGTTCCCCCGCTCAGACC

**Emi1:.**

Forward - GGACTTAATCAATGTGTCTAAAG

Reverse - CTTGGCAACCTCAGAGAATTC

**Cyclin B1.**

Forward - CGGTTCATGCAGAATAATTGTGTGC

Reverse - GTTGCTCGACATCAACCTCTCCAATC

**Plk-1.**

Forward - GGTCAGGCAAGAGGAGGCTG

Reverse - GGAGGGTGATCTTCTTCATC

**Cdc20.**

Forward - GCTGTCAAGGCCGTAGCATGG

Reverse - GATGTGTGACCTTTGAGTTC

**Cyclophilin.**

Forward - GACCCAACACAAATGGTTC

Reverse – AGTCAGCAATGGTGATCTTC

## Supporting Information

Figure S1 **TM downregulates the transcription of Plk-1, cyclin B1, and Cdc20.** HeLa cells were treated with DMSO or 1 µg/ml TM for 16 h. The mRNA levels of the indicated genes were quantified by SYBR-green qRT-PCR.
(TIF)

Figure S2 **Degradation of Cdh1 during ER stress is not APC/C-mediated.** (**A**) HeLa cells transfected with myc-Cdh1 were treated with DMSO or 1 µg/ml of TM. Total cell lysates were collected every 2 h over 10 h and immunoblotted for myc. (B) HeLa cells transfected with empty vector or Cdc27-specific shRNA (Cdc27-KD) were treated with DMSO or 1 µg/ml of TM for the indicated times. Total cell lysates were immunoblotted for the indicated proteins.
(TIF)

Figure S3 **Depletion of Cdh1 overcomes ER stress-induced G1 delay.** (A) DNA content of empty vector-transfected or Cdh1-KD cells treated with DMSO or 2.5 µg/ml TM for 8 h were analyzed by flow cytometry. Representative FACS histograms show cell cycle distribution of DMSO-treated cells (red) and TM-treated cells (blue). (B) DNA content of empty vector-transfected or Cdh1-KD cells treated with DMSO or 1 µg/ml TM for 16 h were analyzed by flow cytometry. Representative FACS histograms are shown. Refer to Figure 2 for quantification of increase in G1 population after TM treatment.
(TIF)

Figure S4 **Cell cycle distribution of cells treated with TM alone or together with the proteasome inhibitor MG-132.** (A) HeLa cells were treated with DMSO or 0.5 µg/ml TM and cell cycle distribution was analyzed by FACS for a sample of these cells. (B) HeLa cells were treated with 0.5 µg/ml TM plus 5 µM MG-132 for 16 h and cell cycle distribution was determined by FACS for a sample of these cells. (C) Quantification of changes in the percentage of G1 and G2/M populations following TM treatment alone (A) or together with MG-132 (B), normalized to the percentage of G1 or G2/M cells in the respective DMSO-treated samples (A). Refer to Figure 3A for the biochemical analysis performed using lysates prepared from these cells.
(TIF)

Figure S5 **APC/C^Cdh1^ does not mediate degradation of Emi1 upon ER stress.** Empty vector-transfected or Cdh1-KD cells were treated with DMSO or 0.5 µg/ml TM for 16 h. Total cell lysates were immunoblotted with the indicated antibodies.
(TIF)

Figure S6 **Overexpression of Emi1 partially rescued ER stress-dependent downregulation of APC/C^Cdh1^ substrates.** Empty vector-transfected or Cdh1-KD cells were treated with DMSO or 2.5 µg/ml TM for 2.5 h, 5 h, and 8 h. Total cell lysates were immunoblotted with the indicated antibodies.
(TIF)

Figure S7 **Cdh1 depletion enhanced susceptibility to ER stress-induced cell death.** Representative FACS histograms of empty vector-transfected and Cdh1-KD cells treated with DMSO or 0.5 µg/ml TM for 9 h, 12 h, and 24 h. Refer to the quantification of sub-G1 (cells with less than 2 N DNA content) in Figure 4D.
(TIF)

Figure S8 **Sensitivity to ER stress-induced cell death in the absence of Cdh1 is not mediated by JNK or CDKs.** Empty vector-transfected or Cdh1-KD cells were treated with DMSO, 0.5 µg/ml TM alone, or 0.5 µg/ml TM plus either 10 µM JNK inhibitor SP600125 (SP) or pan-CDK inhibitor roscovitine (Rosc). Graphs show quantification of sub-G1 population in cells treated with the indicated drugs for 8 h, 12 h, and 24 h by flow cytometry.
(TIF)

Figure S9 **ER stress downregulates the protein level of APC/C^Cdh1^ substrates in HFF-1 cells.** HFF-1 cells were treated with DMSO or 1 µg/ml of TM for 16 h. Total cell lysates were immunoblotted for the indicated endogenous proteins.
(TIF)
