# Supplementary figures and images for: The Anaphase-Promoting Complex or Cyclosome Supports Cell Survival in Response to Endoplasmic Reticulum Stress
Source: PLoS One. 2012 Apr 23;7(4):e35520. doi: 10.1371/journal.pone.0035520 (PMC3335095; doi:10.1371/journal.pone.0035520)

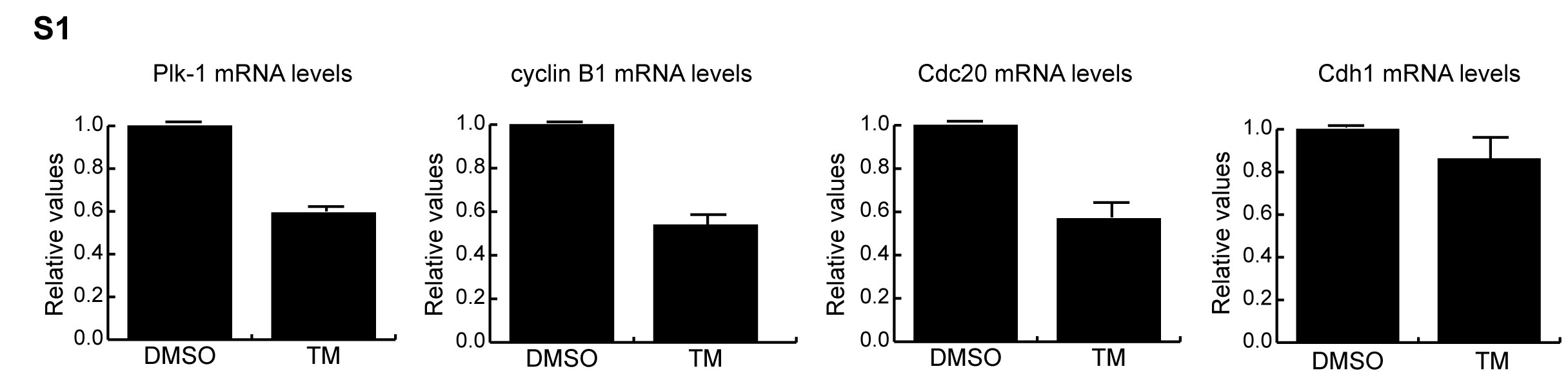

Supplement: Figure S1 — TM downregulates the transcription of Plk-1, cyclin B1, and Cdc20. HeLa cells were treated with DMSO or 1 µg/ml TM for 16 h. The mRNA levels of the indicated genes were quantified by SYBR-green qRT-PCR. (TIF) [file pone.0035520.s001.tif]

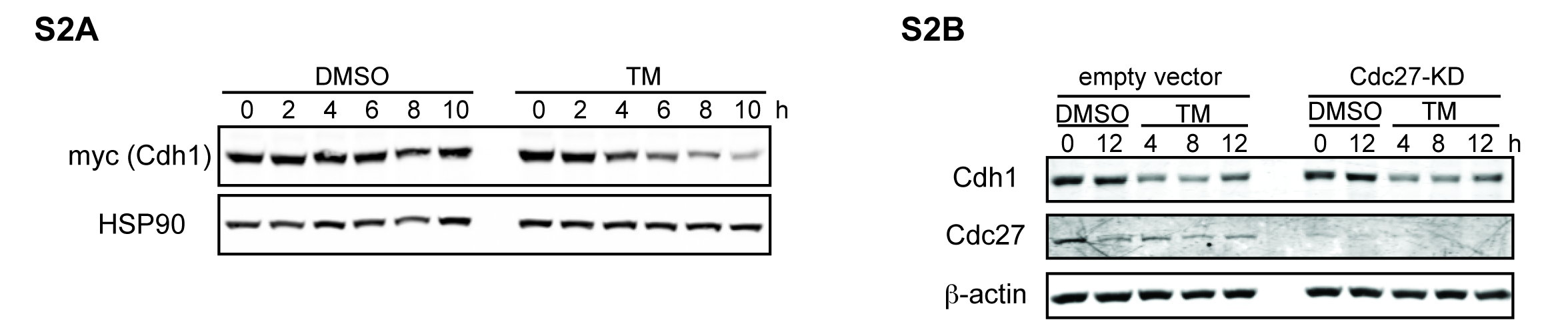

Supplement: Figure S2 — Degradation of Cdh1 during ER stress is not APC/C-mediated. (A) HeLa cells transfected with myc-Cdh1 were treated with DMSO or 1 µg/ml of TM. Total cell lysates were collected every 2 h over 10 h and immunoblotted for myc. (B) HeLa cells transfected with empty vector or Cdc27-specific shRNA (Cdc27-KD) were treated with DMSO or 1 µg/ml of TM for the indicated times. Total cell lysates were immunoblotted for the indicated proteins. (TIF) [file pone.0035520.s002.tif]

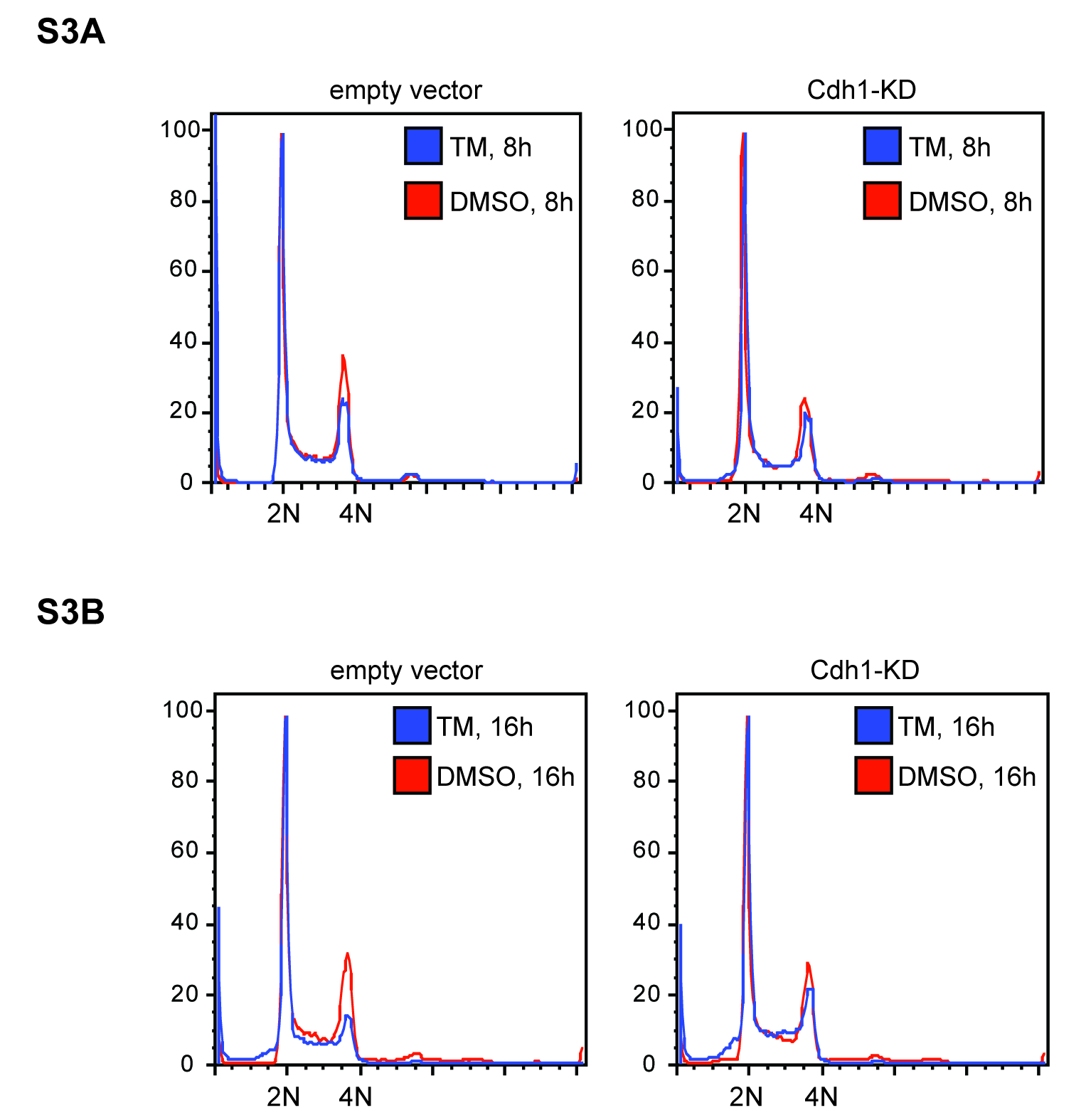

Supplement: Figure S3 — Depletion of Cdh1 overcomes ER stress-induced G1 delay. (A) DNA content of empty vector-transfected or Cdh1-KD cells treated with DMSO or 2.5 µg/ml TM for 8 h were analyzed by flow cytometry. Representative FACS histograms show cell cycle distribution of DMSO-treated cells (red) and TM-treated cells (blue). (B) DNA content of empty vector-transfected or Cdh1-KD cells treated with DMSO or 1 µg/ml TM for 16 h were analyzed by flow cytometry. Representative FACS histograms are shown. Refer to Figure 2 for quantification of increase in G1 population after TM treatment. (TIF) [file pone.0035520.s003.tif]

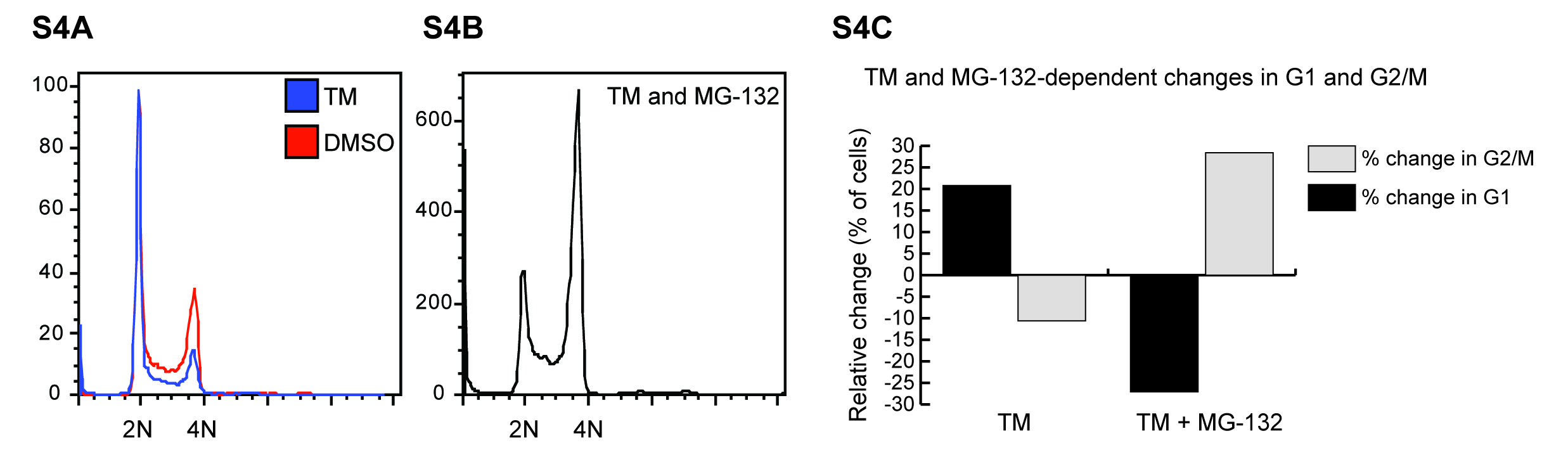

Supplement: Figure S4 — Cell cycle distribution of cells treated with TM alone or together with the proteasome inhibitor MG-132. (A) HeLa cells were treated with DMSO or 0.5 µg/ml TM and cell cycle distribution was analyzed by FACS for a sample of these cells. (B) HeLa cells were treated with 0.5 µg/ml TM plus 5 µM MG-132 for 16 h and cell cycle distribution was determined by FACS for a sample of these cells. (C) Quantification of changes in the percentage of G1 and G2/M populations following TM treatment alone (A) or together with MG-132 (B), normalized to the percentage of G1 or G2/M cells in the respective DMSO-treated samples (A). Refer to Figure 3A for the biochemical analysis performed using lysates prepared from these cells. (TIF) [file pone.0035520.s004.tif]

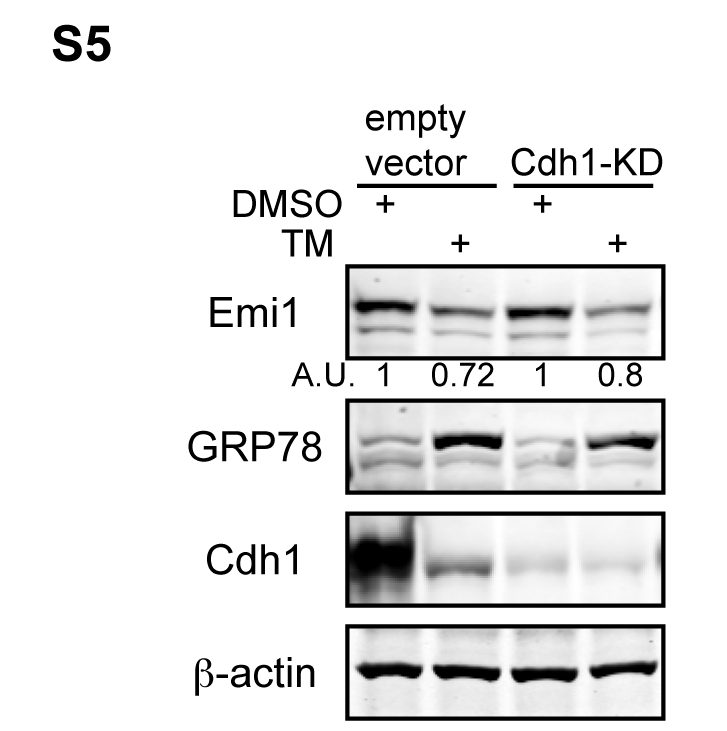

Supplement: Figure S5 — APC/CCdh1 does not mediate degradation of Emi1 upon ER stress. Empty vector-transfected or Cdh1-KD cells were treated with DMSO or 0.5 µg/ml TM for 16 h. Total cell lysates were immunoblotted with the indicated antibodies. (TIF) [file pone.0035520.s005.tif]

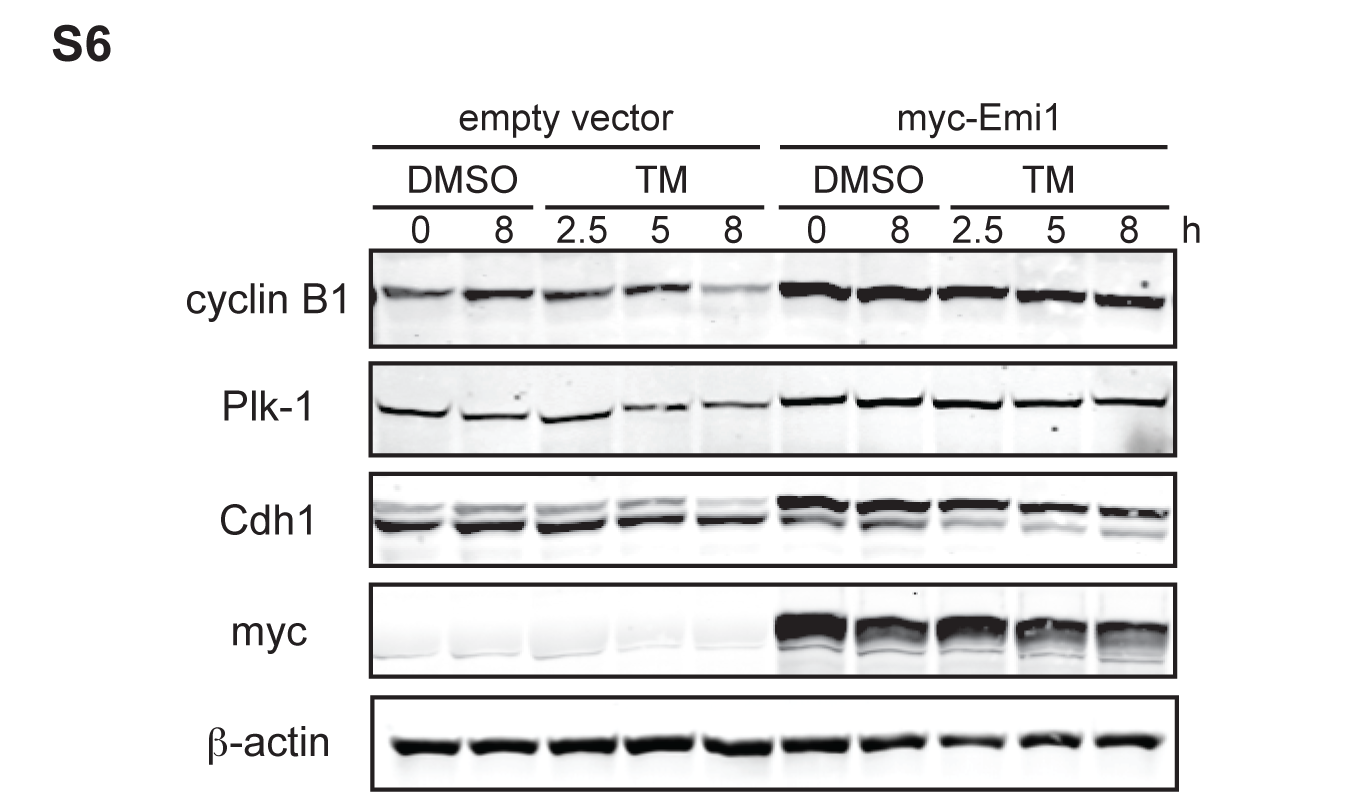

Supplement: Figure S6 — Overexpression of Emi1 partially rescued ER stress-dependent downregulation of APC/CCdh1 substrates. Empty vector-transfected or Cdh1-KD cells were treated with DMSO or 2.5 µg/ml TM for 2.5 h, 5 h, and 8 h. Total cell lysates were immunoblotted with the indicated antibodies. (TIF) [file pone.0035520.s006.tif]

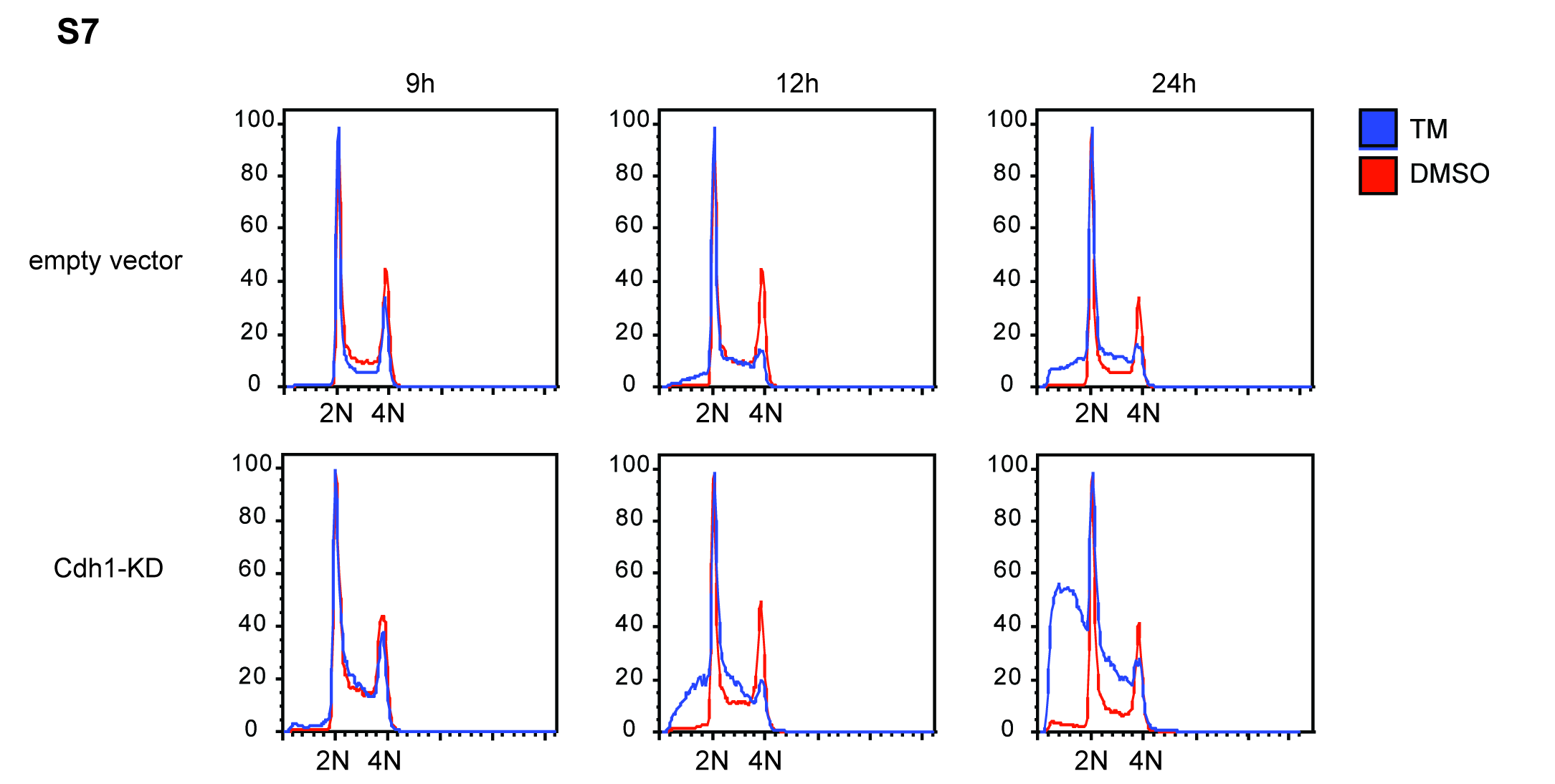

Supplement: Figure S7 — Cdh1 depletion enhanced susceptibility to ER stress-induced cell death. Representative FACS histograms of empty vector-transfected and Cdh1-KD cells treated with DMSO or 0.5 µg/ml TM for 9 h, 12 h, and 24 h. Refer to the quantification of sub-G1 (cells with less than 2 N DNA content) in Figure 4D. (TIF) [file pone.0035520.s007.tif]

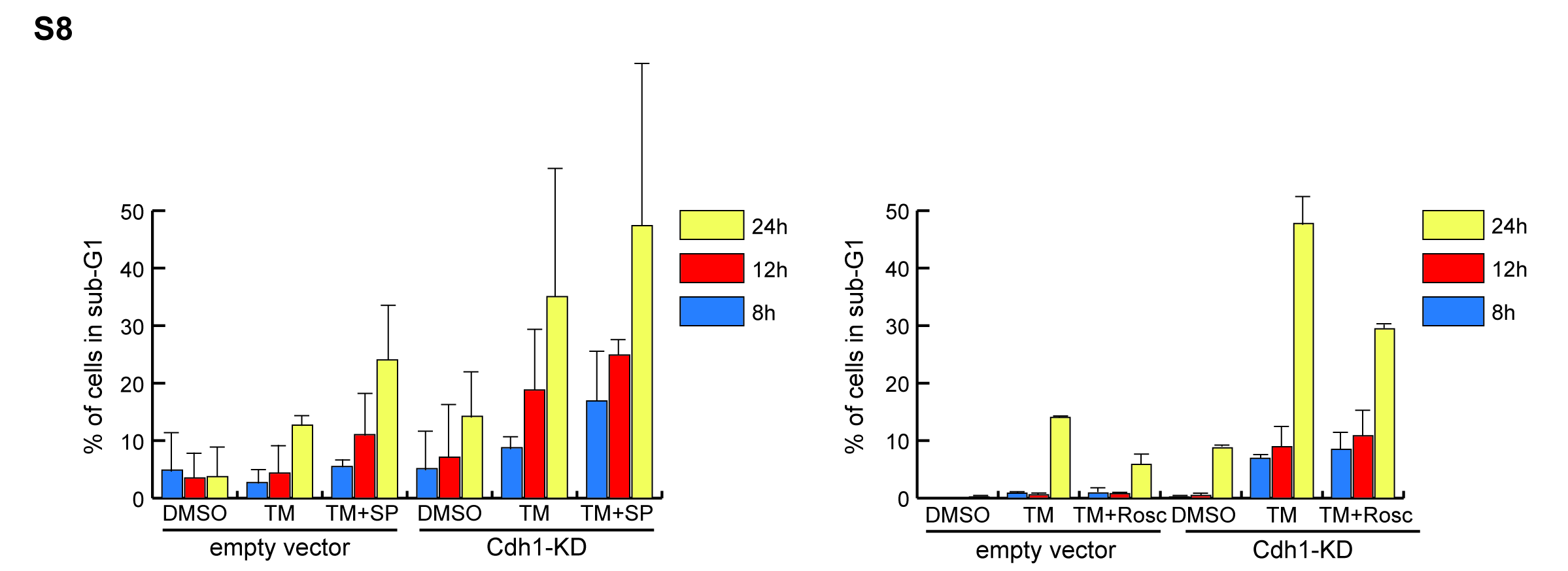

Supplement: Figure S8 — Sensitivity to ER stress-induced cell death in the absence of Cdh1 is not mediated by JNK or CDKs. Empty vector-transfected or Cdh1-KD cells were treated with DMSO, 0.5 µg/ml TM alone, or 0.5 µg/ml TM plus either 10 µM JNK inhibitor SP600125 (SP) or pan-CDK inhibitor roscovitine (Rosc). Graphs show quantification of sub-G1 population in cells treated with the indicated drugs for 8 h, 12 h, and 24 h by flow cytometry. (TIF) [file pone.0035520.s008.tif]

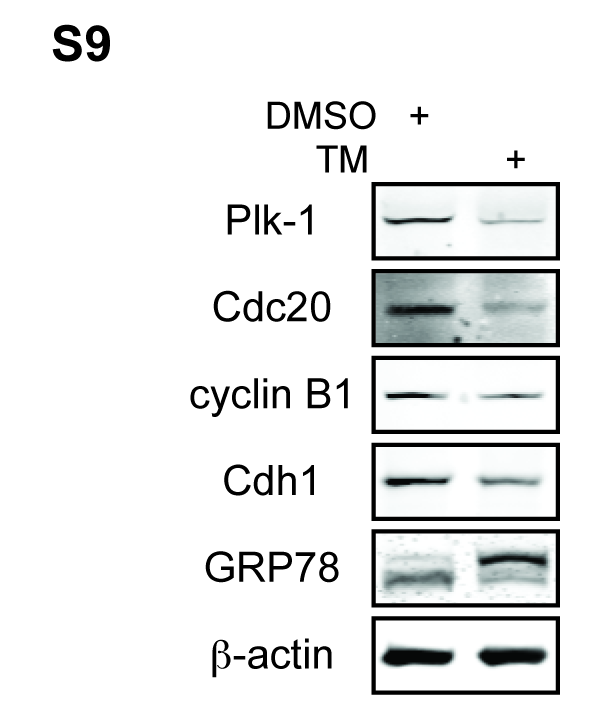

Supplement: Figure S9 — ER stress downregulates the protein level of APC/CCdh1 substrates in HFF-1 cells. HFF-1 cells were treated with DMSO or 1 µg/ml of TM for 16 h. Total cell lysates were immunoblotted for the indicated endogenous proteins. (TIF) [file pone.0035520.s009.tif]
